# From Synapses to Circuits, Astrocytes Regulate Behavior

**DOI:** 10.3389/fncir.2021.786293

**Published:** 2022-01-04

**Authors:** Krissy A. Lyon, Nicola J. Allen

**Affiliations:** Molecular Neurobiology Laboratory, The Salk Institute for Biological Studies, La Jolla, CA, United States

**Keywords:** astrocyte, behavior, chemogenetic, optogenetic, GPCR

## Abstract

Astrocytes are non-neuronal cells that regulate synapses, neuronal circuits, and behavior. Astrocytes ensheath neuronal synapses to form the tripartite synapse where astrocytes influence synapse formation, function, and plasticity. Beyond the synapse, recent research has revealed that astrocyte influences on the nervous system extend to the modulation of neuronal circuitry and behavior. Here we review recent findings on the active role of astrocytes in behavioral modulation with a focus on *in vivo* studies, primarily in mice. Using tools to acutely manipulate astrocytes, such as optogenetics or chemogenetics, studies reviewed here have demonstrated a causal role for astrocytes in sleep, memory, sensorimotor behaviors, feeding, fear, anxiety, and cognitive processes like attention and behavioral flexibility. Current tools and future directions for astrocyte-specific manipulation, including methods for probing astrocyte heterogeneity, are discussed. Understanding the contribution of astrocytes to neuronal circuit activity and organismal behavior will be critical toward understanding how nervous system function gives rise to behavior.

## Introduction

A major goal in neuroscience is to understand how neuronal circuitry maps onto specific physiological functions and behaviors. Neural circuits are recognized as the basis of nervous system function, therefore neuroscience research has largely focused on neurons from the level of the synapse to brain-wide circuits (Tosches, 2017). Yet, the contributions of astrocytes, key players in brain function, have long been overlooked. Astrocytes are glial cells that tile throughout the brain ensheathing neuronal synapses to form the “tripartite synapse,” in which neurons and astrocytes bidirectionally communicate (Araque et al., 1999; Allen and Barres, 2009). The functions of astrocytes at the synapse include clearance of excess neurotransmitter, maintenance of ion homeostasis, and release of neuroactive factors influencing synapse development and function (Allen and Barres, 2009; Murphy-Royal et al., 2017; Farhy-Tselnicker and Allen, 2018). Bidirectional astrocyte-neuron communication includes astrocyte response to synaptic activity via neurotransmitter receptors (Porter and McCarthy, 1997; Kofuji and Araque, 2021) and astrocytic release of factors. Unlike neurons, astrocytes do not fire action potentials, but are capable of release of gliotransmitters (Bezzi and Volterra, 2001; Bezzi et al., 2004; Fiacco and McCarthy, 2004; Jourdain et al., 2007; Navarrete et al., 2013; Araque et al., 2014; Harada et al., 2015) such as ATP (Guthrie et al., 1999; Gourine et al., 2010) and D-serine (Wolosker et al., 1999; Yang et al., 2003; Henneberger and Rusakov, 2010) which can modulate neuronal activity. While the importance of astrocytes for proper synapse formation, function, and plasticity is now appreciated (Farhy-Tselnicker and Allen, 2018; Durkee and Araque, 2019; Sancho et al., 2021; Shan et al., 2021; Wahis et al., 2021b), many questions remain about the capacity of astrocytes to influence behavior.

In this review, we will focus on recent findings of astrocyte influence on behavior including sleep, memory, sensorimotor activity, feeding behavior, fear, anxiety, and additional cognitive processes. Prior to the advent of optogenetic and chemogenetic tools, the study of astrocyte regulation of behavior was hindered by the lack of tools to specifically manipulate astrocytes without also targeting neurons. For example, pharmacological approaches, such as thapsigargin, a SERCA pump inhibitor used to elevate intracellular calcium concentrations (Jackson et al., 1988), might target all cell populations. We will discuss current tools to selectively manipulate astrocytes as well as future directions for further delineation of astrocyte regulation of behavior, especially as relates to understanding astrocyte function based on anatomical location or astrocyte subtypes. Among the extensive literature, this review will highlight the most recent research, particularly *in vivo* studies, of astrocyte influence on behavior in rodents. *In vitro* studies remain critical for elucidation of molecular and cellular mechanisms, but *in vivo* experiments are necessary for understanding the role of astrocytes in whole organismal behavior. Likewise, *in vivo* experiments can address the effects of sensory input and behavioral state on astrocytes themselves (Nimmerjahn, 2009). *In vitro* astrocyte manipulations have been reviewed elsewhere (Bang et al., 2016) as have recent findings including *C. elegans, D. rerio*, and *D. melanogaster* (Nagai et al., 2021a).

### Physiologically Relevant Tools to Manipulate Astrocyte Activity

**FIGURE 1:**
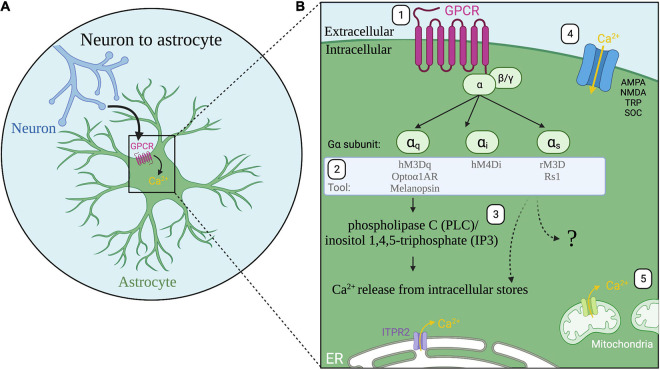
Astrocyte G-protein coupled receptor (GPCR) signaling. **(A)** A major communication pathway from neurons to astrocytes occurs through GPCRs expressed by astrocytes. **(B)** (1) Activation of astrocyte GPCRs (magenta) activate alpha subunits Gαq, Gαi, or Gαs. (2) Tools for astrocyte manipulation target these pathways. (3) Following Gq GPCR activation, the phospholipase C (PLC)/inositol 1,4,5-triphosphate (IP3) pathway and ITPR2 receptor activation induces release of calcium (Ca^2+^, yellow) from the endoplasmic reticulum (ER, white). All three G protein signaling pathways are known to increase calcium in astrocytes though the intracellular signaling pathways are not fully resolved (question mark). (4) Additional sources of calcium include calcium influx through ionotropic glutamate receptors α-amino-3-hydroxy-5-methyl-4-isoxazolepropionic acid receptor (AMPA) and N-methyl-d-aspartate receptor (NMDA); transient receptor potential (TRP) channels; store-operated calcium channels (SOC); and reversed operation of the Na^+^/Ca^2+^ exchanger (not shown). (5) Calcium is also released from mitochondria.

Astrocyte excitability is primarily mediated through calcium signaling (Agulhon et al., 2008; Nimmerjahn, 2009; Zorec et al., 2012) which is complex and varies in duration, amplitude, event rate, and cellular localization of calcium events (Shigetomi et al., 2008, 2010; Bazargani and Attwell, 2016; Wang et al., 2019). Astrocytes display dynamic intracellular calcium signaling which can be spontaneous or triggered by neuronal activity (Cornell-Bell et al., 1990; Nett et al., 2002; Khakh and McCarthy, 2015; Bazargani and Attwell, 2016). One of the main communication pathways between neurons and astrocytes is through G-protein coupled receptors (GPCRs) expressed by astrocytes (Figure 1A; Neves et al., 2002; Kofuji and Araque, 2021). GPCRs interact with heterotrimeric G proteins alpha (α), beta (β), and gamma (γ) (Figure B). Following GPCR activation, the alpha subunit, subdivided into Gq, Gi, and Gs families, dissociates from the beta and gamma proteins leading to downstream signaling cascades including the phospholipase C (PLC)/inositol 1,4,5-triphosphate (IP3) pathway which results in calcium release from the endoplasmic reticulum (Figure 1B; Yu et al., 2020; Kofuji and Araque, 2021). *In vivo* fluorescent calcium imaging revealed correlations of astrocyte calcium activity with visual responses (Schummers et al., 2008), locomotion (Dombeck et al., 2007; Nimmerjahn et al., 2009) and cortical oscillations (Poskanzer and Yuste, 2016) indicating a link between astrocyte calcium activity, neuronal circuits, and behavior. While astrocyte intracellular calcium signaling is critical to astrocyte-neuron communication, the functional relevance of different features of calcium signaling remains a topic of exploration (Bazargani and Attwell, 2016; Guerra-Gomes et al., 2017).

**FIGURE 2:**
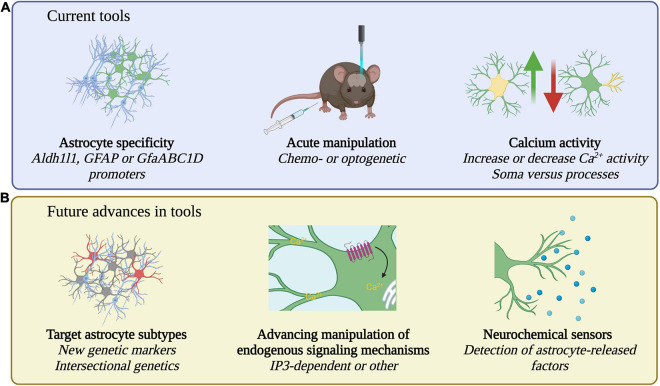
Current and future tools for the study of astrocytes. **(A)** Current tools employ Aldh1ll1, GFAP, or GfaABC1D promoters to specifically target astrocytes. Acute manipulations including chemo- or optogenetic approaches and bidirectional increase or decrease of calcium activity, in soma or processes, are used to probe causal relationships between astrocytes and behavior. **(B)** Future research may identify new genetic markers for astrocytes, strategies for subtype specific manipulation, manipulations that target additional endogenous signaling pathways, and may detect astrocyte released factors with neurochemical sensors.

Many recent studies pair *in vivo* calcium imaging with effector tools to simultaneously image astrocyte or neuron calcium activity alongside astrocyte-specific perturbations (see Table 1 for summary of tools) (Gourine et al., 2010; Chen et al., 2016; Asrican et al., 2020; Corkrum et al., 2020; Ung et al., 2020; Vaidyanathan et al., 2021). For cell type specificity, astrocytes are primarily targeted by expressing effectors under the control of Aldh1l1 (Tsai et al., 2012), GFAP (Lee et al., 2008), or the shortened GfaABC1D promoters (Shigetomi et al., 2013; Figure 2A), either through viral vector delivery or in transgenic rodents. Both optogenetic and chemogenetic tools have been applied in astrocytes but experimental manipulation of astrocytes comes with additional caveats (Mu et al., 2019). Astrocytes are not neurons and tools designed for use in neurons must be carefully characterized in astrocytes before assessing behavioral changes (Figueiredo et al., 2014; McNeill et al., 2021). For example, the light-sensitive cation channel channelrhodopsin is widely used to activate neurons through the generation of action potentials (Boyden et al., 2005). Yet, studies have employed channelrhodopsin in astrocytes which do not fire action potentials. Stimulation of channelrhodopsin in astrocytes is used to increase intracellular calcium, but this also leads to an increase in extracellular potassium (Octeau et al., 2019). While the mechanism of increased potassium is unknown, such increases could impact neuronal firing and confound behavioral results (Octeau et al., 2019; Rasmussen et al., 2019) highlighting the importance of careful characterization of tools in astrocytes. Given the highly prevalent expression of GPCRs in astrocytes, tools targeting GPCR signaling pathways may be more physiologically relevant. However, it is important to note that Gi-coupled DREADDs (Designer Receptors Exclusively Activated by Designer Drugs) (Alexander et al., 2009; Roth, 2016) employed to inhibit neurons have the opposite effect on astrocytes and increase calcium activity and promote gliotransmitter release (Chai et al., 2017; Durkee et al., 2019; Figure 1). Lastly, astrocytes also contact other astrocytes via gap junctions (Rose and Ransom, 1997), so studies aiming to manipulate subsets of astrocytes must also consider the interconnectedness of astrocytes (McNeill et al., 2021).

**TABLE 1:** Tools used to probe astrocyte influence on behavior.

Tool	Description	Stimulus/ligand	Effect in astrocytes
**Optogenetics**			
Channelrhodopsin	Light-sensitive cation channel	Blue light	Increases intracellular Ca^2+^ in astrocytes Gourine et al. (2010) but also increases extracellular K + Octeau et al. (2019)
Archaerhodopsin	Light-sensitive outward proton pump	Green-yellow light	Increases intracellular Ca^2+^ in astrocytes Poskanzer and Yuste (2016)
Optoα1AR	Light-sensitive Gq-coupled receptor	Blue light	Increases intracellular Ca^2+^ in astrocytes Adamsky et al. (2018), for mouse line see Iwai et al. (2021)
Melanopsin (Opn4)	Gα_*q–11*_-coupled photopigment	Blue light	Increases intracellular Ca^2+^ in astrocytes Mederos et al. (2019)
Mlc1-bPAC	Photoactivated cyclase from *Beggiatoa* bacterium	Blue light	Elevates astrocyte cAMP Zhou et al. (2021)
**Chemogenetics**			
hM3Dq	Gq-GPCR coupled DREADD	CNO	Increases intracellular Ca^2+^ in astrocytes Agulhon et al. (2013)
hM4Di	Gi-GPCR coupled DREADD	CNO	Increases intracellular Ca^2+^ in astrocytes Chai et al. (2017), Durkee et al. (2019)
rM3D	Gs-GPCR coupled DREADD	CNO	Increases intracellular Ca^2+^ in astrocytes Chai et al. (2017)
Rs1	Gs-GPCR coupled mutated serotonin receptor	GR-125487	Increased cAMP in cultured astrocytes; Some ligand-independent constitutive Gs-coupled activity Orr et al. (2015)
**Other**			
*Itpr2−/−* mouse line	Knockout of Ca^2+^ channel activated by inositol trisphosphate	NA	Reduces GPCR mediated Ca^2+^ elevations Petravicz et al. (2008)
DN-SNARE mouse line	Dominant-negative domain of vesicular SNARE		Alters astrocyte vesicular trafficking and release Pascual et al. (2005) but see also Fujita et al. (2014)
VIPP mouse line	Overexpression of venus tagged Ins(1,4,5)P_3_5′phosphatase (IPP)		Metabolizes IP3, reduced Ca^2+^ signaling in astrocytes Foley et al. (2017)
p130PH	Overexpression of domain of phospholipase C (PLC)-like protein p130		Buffers cytosolic IP3 to inhibit release of Ca2 + from internal stores Xie et al. (2010)
iβARK	RGS domain of β-adrenergic receptor kinase 1		Sequesters Gαq-GTP, reducing Gq GPCR Ca^2+^ signaling Nagai et al. (2021b)
CalEx	Modified human plasma membrane Ca^2+^ pump PMCA2		Reduces astrocyte Ca^2+^ signaling Yu et al. (2018)
Calcium indicators	Typically fluorescence emission following Ca^2+^ binding		Not a manipulation but a readout for calcium activity

*Description of tools used to manipulate astrocytes, including details on astrocyte-specific effects.*

### Astrocyte Regulation of Behavior

Early evidence for astrocyte regulation of behavior emerged when Halassa et al. (2009) manipulated astrocyte function and observed altered sleep (discussed below in *Sleep* section). Next, chemosensitive hindbrain astrocytes were shown to modulate respiration in rats (Gourine et al., 2010). In response to decreases in pH, corresponding to elevated blood CO_2_ levels, astrocyte intracellular calcium levels rise leading to increased astrocytic ATP release, chemoreceptor neuron activation, and increased adaptive breathing revealing a role for astrocytes in a critical homeostatic reflex. Other studies showed that activation of astrocyte Gq-GPCR signaling using hM3Dq DREADD increased heart rate and blood pressure, while decreasing body temperature, indicating astrocyte involvement in additional homeostatic processes (Agulhon et al., 2013). Over the past decade, a role for astrocytes in behavior modulation has become increasingly clear.

### Sleep

Based upon his histological and morphological studies, Ramon y Cajal hypothesized that astrocytes control the switch from wakefulness to sleep (Garcia-Marin et al., 2007). Modern tools for astrocyte-specific manipulation have recently allowed researchers to probe neuron-astrocyte interactions in the regulation of sleep and wake *in vivo*. Using a mouse line expressing a dominant negative SNARE protein in astrocytes (DN-SNARE) (Pascual et al., 2005; Fujita et al., 2014) that alters astrocyte vesicular trafficking and release, Halassa et al. (2009) demonstrated a role for astrocytes in sleep pressure, or the drive to sleep following wakefulness. This was further shown to be in an adenosine A1 receptor-dependent manner linking astrocyte-derived adenosine to sleep regulation. Later studies outlined below employed optogenetic activation of astrocytes to acutely test a role in sleep.

Neuronal activity changes in different phases of sleep. During rapid eye movement (REM) sleep neural oscillations are fast and desynchronized while neuronal activity in non-REM (NREM) sleep is synchronized with high-amplitude, low-frequency oscillations also referred to as slow wave sleep (Maquet, 2001). Channelrhodopsin activation of astrocytes in the anterior cingulate cortex of mice reduces NREM sleep in the light period (Yamashita et al., 2014) while activation of posterior hypothalamus astrocytes increases REM and NREM sleep in the dark period (Pelluru et al., 2016), indicating possible region-specific, or circadian time-dependent, roles for astrocytes in the regulation of sleep. Indeed, suprachiasmatic nucleus astrocytes display increased calcium signaling at night (Brancaccio et al., 2017) and can drive circadian behavior in mice through glutamate release (Brancaccio et al., 2019). Lifelong astrocyte-specific knockout of connexin 43, a gap junction component, also increases sleep in the dark period by silencing orexin wake neurons (Clasadonte et al., 2017). *In vivo* two-photon calcium imaging allowed researchers to correlate astrocyte calcium activity with features of sleep. For example, astrocyte calcium activity increases with sleep need in *D. melanogaster* (Blum et al., 2021). Additionally, increases in cortical astrocyte calcium precede shifts to slow wave activity, or the highly synchronized firing of neurons, in mice (Poskanzer and Yuste, 2016). While this latter study was conducted in anesthetized mice, this type of synchronized neuronal firing is typically indicative of slow wave sleep. Increasing astrocyte calcium activity through optogenetic activation shifted the local neuronal network to the synchronized state, suggesting an active role for astrocytes in cortical circuit dynamics (Poskanzer and Yuste, 2016).

Recently, calcium imaging in awake-behaving mice has enabled the study of natural sleep (Bojarskaite et al., 2020; Vaidyanathan et al., 2021). Tracking cortical astrocyte calcium activity across sleep and wake, Bojarskaite et al. (2020) found astrocyte calcium activity is lowest during sleep, increases during wake activities including whisking and locomotion, and is highest in the transition from sleep to wake. In this same study, *Itpr2* (alias *Ip3r2*) knockout mice, which have attenuated astrocyte calcium signaling throughout the brain (Petravicz et al., 2008), did not show increased calcium upon awakening and had disrupted slow wave sleep (NREM and intermediate state sleep) linking astrocyte calcium activity to slow wave activity in natural sleep. Earlier studies found that mice with overexpression of Ins(1,4,5)P_3_ 5′-phosphatase (IPP) in astrocytes (Table 1), had attenuated calcium signaling in hippocampal astrocytes but largely normal calcium signaling in the cortex and normal NREM sleep (Foley et al., 2017), potentially indicating a specific role for cortical astrocytes in NREM sleep regulation.

Studies to increase astrocyte intracellular calcium using chemogenetic activation of cortical astrocyte Gi-GPCR signaling showed increased slow wave activity, whereas activation of the Gq-GPCR pathway disrupted sleep-wake transitions (Vaidyanathan et al., 2021). Thus, astrocytes regulate distinct features of natural sleep through separable signaling pathways. As noted above, both Gi and Gq signaling increase intracellular calcium in astrocytes (Mariotti et al., 2016; Durkee et al., 2019) thus this study shows that two different GPCR signaling pathways that both lead to increased calcium can lead to different behavioral outputs (Vaidyanathan et al., 2021). Existing evidence for astrocyte regulation of sleep primarily points to a role in the regulation of slow wave/NREM sleep. Future studies may continue to parse how distinct astrocyte calcium signaling pathways, and specific elements of calcium signaling such as cellular localization, duration, and amplitude (Wang et al., 2019), regulate different features of sleep. Beyond regulation of sleep-wake states, astrocytes promote solute clearance from the brain during sleep (Xie et al., 2013; Haydon, 2017) and astrocyte influences likely extend to the memory consolidation role of sleep, further discussed below.

### Memory

Changes in sleep have the potential to impact other behaviors as memory consolidation is considered one important function of sleep (Stickgold, 2005; Abel and Klann, 2013; Klinzing et al., 2019). In discussing astrocytes and memory, we will briefly focus on known roles for astrocytes in both sleep and memory and then focus solely on memory. As discussed above, *Itpr2* knockout mice have blunted astrocyte calcium signaling and altered NREM sleep (Bojarskaite et al., 2020). Pinto-Duarte et al. (2019) found *Itpr2* knockout mice also have deficient remote memory recall but unaffected learning and short-term memory. While brain-wide reduction of astrocyte calcium signaling disrupted NREM sleep and remote memory in separate studies, elevated cortical astrocyte calcium signaling increased delta oscillations which are associated with the weakening of memories (Vaidyanathan et al., 2021) indicating that astrocytes may influence both the strengthening and weakening of memories. While those studies did not test both sleep and memory regulation by astrocytes, Halassa et al. (2009) found impaired novel objection recognition following sleep deprivation in wildtype mice but DN-SNARE mice did not display this deficit. Thus, gliotransmission may regulate memory deficits following sleep deprivation.

Astrocyte secreted proteins and gliotransmitters shape neuronal plasticity (Santello et al., 2019; Sancho et al., 2021). Synaptic plasticity, including long term potentiation, underlies memory and astrocyte-released factors involved in these processes include D-serine (Yang et al., 2003; Panatier et al., 2006; Henneberger and Rusakov, 2010) and ATP/adenosine (Florian et al., 2011). Mice lacking these factors or their release from astrocytes display altered memory (Halassa et al., 2009; Florian et al., 2011; Lee et al., 2014). The contributions of astrocyte secreted factors and gliotransmission to memory are reviewed in detail elsewhere (Ben Achour and Pascual, 2012; De Pitta et al., 2016). Here, we will focus on recent work with acute manipulation of astrocytes, using optogenetic and chemogenetic tools, to probe astrocyte regulation of memory across phases of acquisition, consolidation, and retrieval. Different types of memory such as spatial memory or long-term memory can be tested in rodents and well-validated assays include the Y- and T-mazes, novel object recognition and placement tests, Barnes maze, Morris water maze, contextual fear conditioning, and other cued memory assays (Swonger and Rech, 1972; Barnes, 1979; Vorhees and Williams, 2014; Wolf et al., 2016).

Many studies have used activation of the Gq-GPCR signaling pathway to test the role of astrocytes in memory. Chemogenetic activation of Gq-GPCR signaling in hippocampal astrocytes improved spatial memory and increased freezing in a contextual fear conditioning assay (Adamsky et al., 2018). Importantly, this Gq-GPCR activation was sufficient to generate *de novo* long term potentiation of CA3 to CA1 synapses thought to underly memory formation (Adamsky et al., 2018). Auditory-cued memory was not affected indicating the engagement of hippocampal astrocyte Gq signaling in select types of memory. Further, increased freezing depended upon Gq activation during memory acquisition or early consolidation but was not affected by activation during memory recall (Adamsky et al., 2018) indicating a role for astrocyte Gq signaling in different phases of memory.

In the anterior cingulate cortex, Gq activation of astrocytes by the light inducible Gq-GPCR, Optoα1AR (Table 1), increased long-term object recognition but did not affect Y-maze or novel object recognition assays (Iwai et al., 2021), suggesting a role for cortical astrocytes in long-term memory but not short-term memory. In the prefrontal cortex (PFC), GABA_B_ receptor knockout in astrocytes reduces low-gamma oscillation power and decreases performance in the T-maze (Mederos et al., 2021). Stimulation of a melanopsin GPCR tool coupled to Gq_11_ (Mederos et al., 2019; Table 1) in astrocytes rescues this deficit while Gq_11_ activation in PFC astrocytes in wildtype mice increases low-gamma oscillation power and improves T-maze performance (Mederos et al., 2021); thus astrocyte Gq signaling may have a bidirectional ability to modulate memory. The recently developed iβARK tool attenuates astrocyte Gq-induced calcium activity (Nagai et al., 2021b). Brain-wide expression of iβARK reduces performance in the Y-maze and the modified novel object placement test (Nagai et al., 2021b). Here, contextual fear conditioning and cued memory are unaffected (Nagai et al., 2021b) suggesting effects only on specific types of memory. While differences in tools used, timescales of activation or attenuation, brain region, and behavioral assays employed make direct comparisons difficult, these recent studies indicate that Gq signaling is indeed critical to astrocyte modulation of memory and hint at regional differences in astrocyte modulation of circuits and behavior.

Fewer studies have investigated the role of astrocyte Gi-GPCR signaling in regulation of memory. Dorsal hippocampal Gi activation attenuates stress-enhanced fear learning (Jones et al., 2018), while CA1 astrocyte Gi activation impairs memory recall and inhibits CA1 to anterior cingulate cortex projections important to memory recall, but not recent memory (Kol et al., 2020). Gi-GPCR signaling is the endogenous signaling pathway activated in astrocytes by μ-opioid receptor agonism and Gi signaling in CA1 astrocytes is implicated in memory acquisition in conditioned place preference (Nam et al., 2019) suggesting the presence of astrocyte subtypes involved in different types of memory within the hippocampus.

Lastly, astrocyte Gs-GPCR signaling affects memory. Typically, Gs stimulates adenylyl cyclase and increases the second messenger cyclic AMP (cAMP) (Neves et al., 2002). In hippocampal and striatal astrocytes, Gs signaling increases calcium signaling though to a lesser extent than Gq signaling (Chai et al., 2017). Activation of Rs1, a modified Gs-coupled receptor (Table 1), in hippocampal and thalamic astrocytes impairs spatial memory in the Morris water maze when activated during training or one day later (Orr et al., 2015). Conversely, elevating hippocampal astrocyte cAMP through a photoactivated adenylyl cyclase (Table 1) during or immediately after training improved performance in a different memory test, the novel object placement test (Zhou et al., 2021). In this same study, increasing cAMP one day after training impaired memory retention (Zhou et al., 2021). Differences in the effect on memory may stem from different memory assays used or different timescales of activation. Orr et al. (2015) report some ligand-independent constitutive Gs activity which reduced novel object recognition, even in the absence of chemogenetic ligand. Long-term Gs activation in hippocampal astrocytes may generally impair memory while acute cAMP increases seem to improve different aspects of memory. Zhou et al. (2021) speculate that increased cAMP during memory formation improves performance through hippocampal neuron long term potentiation but that increased cAMP the following day could reduce the original memory through the same mechanism akin to “re-writing” or replacing the memory. Memory is an energetically demanding process, and in addition to the roles discussed here, astrocytes also influence memory by providing energy to neurons through astrocyte-neuron metabolic coupling (Suzuki et al., 2011; Alberini et al., 2018).

### Sensory and Motor

Astrocytes respond to sensory and motor activity through elevations in intracellular calcium signaling. For example, astrocyte calcium signaling increases in response to the stimulation of whiskers, in a frequency-dependent manner (Wang et al., 2006), noting this study was conducted in anesthetized mice which may have altered astrocyte activity. Advances in two-photon imaging with stable head-mounted microscopes allowed for imaging of astrocyte calcium activity in awake behaving mice (Dombeck et al., 2007). Using two-photon imaging with cellular resolution, Dombeck et al. (2007) found calcium activity correlated with running in a subset of sensory cortex astrocytes in awake behaving mice. Similar techniques revealed increased barrel cortex astrocyte endfoot calcium activity in response to natural hyperemia during whisking or locomotion (Tran et al., 2018), demonstrating astrocyte calcium responses to vascular signaling dependent on behavioral state.

In the cerebellum, large networks of radial astrocytes called Bergmann glia display increased calcium activity at the onset of locomotion (Nimmerjahn et al., 2009). Channelrhodopsin stimulation of Bergmann glia increases the horizontal optokinetic reflex and pupil size, both known to be modulated by cerebellar circuits (Sasaki et al., 2012), suggesting a role for Bergmann glia in visual reflexes. Channelrhodopsin activation of V1 astrocytes altered the visual response properties of local neurons affecting both local parvalbumin and somatostatin interneuron excitability (Perea et al., 2014). Calcium signaling in visual cortex astrocytes shows responsiveness to visual stimuli (Schummers et al., 2008). Thus, astrocyte calcium activity correlates with behavioral outputs and manipulation of astrocyte activity alters sensory behavior.

More recently, astrocyte calcium imaging paired with electrocorticogram monitoring of neuronal activity revealed that somatosensory cortex astrocytes respond to sensory stimuli in a stimulus-dependent manner in anesthetized mice (Lines et al., 2020). Application of an electrode to the hindpaw induces cortical neuron gamma activity, thought to underlie cognitive processes, followed by increased astrocyte calcium activity that appears to tamp down neuronal gamma activity. This neuronal gamma activity remained high in *Itpr2* knockout mice with blunted astrocyte calcium signaling indicating astrocyte regulation of sensory-evoked neuronal activity. Likewise, astrocyte Gq activation by hM3Dq DREADD reduced sensory-evoked neuronal gamma activity (Lines et al., 2020). DN-SNARE mice also show reduced cortical gamma oscillations (Lee et al., 2014) suggesting a role for gliotransmitters. Lastly, astrocytes may also modulate tactile discrimination. In the ventrobasal nucleus of the thalamus, astrocytes are the reported source of tonic GABA inhibition important to tactile discrimination. Local infusion of GABA reduces tactile acuity while astrocyte-specific knockdown of GABA synthesis pathway genes increased tactile acuity (Kwak et al., 2020). Therefore astrocytes regulate neuronal responses to sensory stimuli both within localized circuits and at the level of cortical neuron networks.

### Feeding

Astrocytes actively regulate metabolic homeostasis and feeding behavior. Changes in diet or fasting alter astrocyte gene expression, morphology, and neuronal ensheathment and astrocytes are responsive to glucose and the hormones insulin, leptin, and ghrelin (Hsuchou et al., 2009; Horvath et al., 2010; Fuente-Martin et al., 2012; Kim et al., 2014; Buckman et al., 2015; Garcia-Caceres et al., 2016, 2019; Varela et al., 2021). Hypothalamic neurons regulate feeding behavior and agouti-related peptide (AgRP) neurons of the arcuate nucleus (ARC) in the ventral floor of the mediobasal hypothalamus (MBH) drive feeding behavior (Wu and Palmiter, 2011; Andermann and Lowell, 2017). However, chemogenetic manipulation of MBH astrocytes has yielded conflicting results. In one study, hM3Dq DREADD activation of MBH astrocytes reduced basal food intake as well as feeding induced by the orexigenic hormone ghrelin (Yang et al., 2015). Conversely, Chen et al. (2016) found hM3Dq DREADD activation of ARC astrocytes increased food intake. As AgRP neuron activation is known to increase feeding behavior (Aponte et al., 2011; Krashes et al., 2011), both studies queried the effect of astrocyte Gq-activation on AgRP neuron activity. Yang et al. (2015) reported astrocyte inhibition of AgRP neurons via adenosine while Chen et al. (2016) reported facilitation of AgRP neurons by ARC astrocytes. In the latter study, astrocyte-mediated increase in food intake was dependent upon AgRP neuron activity as hM4Di inhibition of AgRP neurons alongside astrocyte activation did not increase food intake. These discrepancies could stem from targeting of the entire MBH (Yang et al., 2015) versus specifically the ARC (Chen et al., 2016) and may indicate region-specific roles for astrocytes in modulation of food intake.

Interestingly, Gi-coupled hM4Di signaling in MBH astrocytes increased and prolonged ghrelin-evoked feeding but did not affect basal feeding (Yang et al., 2015) noting that hM4Di signaling has more recently been shown to increase astrocyte calcium signaling and promote gliotransmitter release (Chai et al., 2017; Durkee et al., 2019), in contrast to inhibition observed in neurons. Reducing astrocyte calcium signaling through buffering of IP3 by p130PH (Xie et al., 2010; Table 1) reduced food intake (Chen et al., 2016). Chen et al. (2016) saw no difference in food intake with hM4Di signaling suggesting that different GPCR signaling pathways within hypothalamic astrocytes may have different behavioral effects, as in the cortex (Vaidyanathan et al., 2021).

The above discrepancies may also arise from food intake analyses across different phases of the light-dark cycle or differing CNO dosage (5 mg/kg vs. 0.3 mg/kg). It is important to note that both studies used the GFAP promoter to target DREADD expression to hypothalamic astrocytes which also targets tanycytes lining the third ventricle, that are also implicated in feeding and energy homeostasis (Bolborea and Dale, 2013; Chen et al., 2016). An additional caveat is neither study assayed additional behaviors such as locomotor or anxiety-like behavior, which may affect feeding behavior and are also modulated by AgRP neurons (Dietrich et al., 2015; Padilla et al., 2016). Optogenetic activation of MBH astrocytes reduced food intake as well as fasting- or ghrelin-induced increases in food intake, also in an A1-receptor dependent manner (Sweeney et al., 2016). Here, locomotor or anxiety-like behavior were assayed but were not affected by optogenetic stimulation of MBH astrocytes.

While the differing roles of ARC versus all MBH astrocytes in feeding and their effects on AgRP neurons remain to be further elucidated, recent research corroborates a role for ARC astrocytes in facilitation of AgRP neuron excitement (Varela et al., 2021). AgRP neuron release of GABA promotes increased glial ensheathment of AgRP neurons resulting in increased AgRP neuron excitability and thus a feed-forward loop driving AgRP neuron excitability (Varela et al., 2021). While this work does not directly test behavior, increases in AgRP neuron excitability are expected to increase feeding behavior (Aponte et al., 2011; Krashes et al., 2011). Astrocytes are additionally implicated in satiety signals. Astrocytes in the dorsal vagal complex are activated after 12 hours of feeding on a high fat diet. Chemogenetic activation of these astrocytes via hM3Dq expression reduced basal and post-fasting food intake (MacDonald et al., 2020). Future *in vivo* studies may employ simultaneous monitoring of neuronal and astrocyte calcium signaling during feeding behavior, alongside astrocyte manipulations, to further resolve the role of astrocytes in feeding in a region-specific manner.

### Fear and Anxiety

Both the hippocampus and the amygdala are important for fear and anxiety (Davis, 1992; Maren, 2008). As discussed in the Memory section, increased Gq signaling in hippocampal astrocytes promoted freezing in the contextual fear conditioning assay (Adamsky et al., 2018) while brain-wide reduction of Gq signaling did not alter freezing (Nagai et al., 2021b). Gi-GPCR activation in the dorsal hippocampus attenuated stress-enhanced fear learning (Jones et al., 2018). In the amygdala, astrocytes modulate fear responses through regulation of synapses in the medial subdivision of the central amygdala (CeM) (Martin-Fernandez et al., 2017). Gq DREADD activation of CeM astrocytes reduced local neuron firing rate and decreased fear expression while anxiety-like behavior, as measured by the elevated plus maze, was unaffected (Martin-Fernandez et al., 2017). Knockdown of astrocyte glucocorticoid receptors in the central amygdala attenuates fear memory and reduces anxiety-like behavior in the open field test (Wiktorowska et al., 2021). Basolateral amygdala astrocytes are also implicated in the modulation of fear extinction, and chemogenetic Gq-GPCR activation facilitates fear extinction (Shelkar et al., 2021). Future studies are required to further delineate the role of astrocytes in fear and anxiety though current evidence indicates amygdala astrocyte activity may reduce fear behavior, while hippocampal astrocyte activity has variable effects, potentially dependent on different GPCR signaling pathways or region. A subset of astrocytes in the lateral and capsular part of the central amygdala (CeL) express oxytocin receptors (OTR) and respond to oxytocin signaling with increased calcium signaling that propagates to neighboring astrocytes via gap junctions (Wahis et al., 2021a). Downstream neuronal NMDA receptor activation increases CeL interneuron excitability ultimately increasing inhibitory input onto medial central amygdala projection neurons. Behaviorally, OTR signaling in these astrocytes has an anxiolytic effect both in pain-free conditions and in a model of neuropathic pain (Wahis et al., 2021a) indicating a role for OTR-expressing astrocytes of the amygdala in anxiety. Future studies are required to understand how other astrocyte subtypes of the CeL influence behavior and, more generally, how interactions of different astrocyte subtypes affect local circuity and downstream behavior.

### Cognitive Processes

Understanding of neuronal regulation of cognitive functions like learning, memory, attention, and decision-making has greatly advanced in the last two decades (Roth and Ding, 2020) yet the role of astrocytes in cognitive processes remains relatively unknown. Memory (discussed above) is the most well-studied, and in this last section we will discuss a series of recent studies demonstrating astrocyte contributions to other aspects of cognitive functioning.

Behavioral flexibility is critical to achieving a goal as it allows an organism to pivot to an alternative strategy or to stop the current behavior and preserve energy after repeated failures. In zebrafish, radial astrocytes modulate these adaptative behavioral responses. With increasing failed attempts at swimming, noradrenergic neurons signal to astrocytes which accumulate this “failure signal” as increasing intracellular calcium. Astrocytes then activate GABA neurons that drive cessation of swimming (Mu et al., 2019). In mice, noradrenaline is also implicated in “priming” astrocytes to respond to changes in cortical neuron network activity (Paukert et al., 2014) indicating that astrocyte networks respond to behavioral state.

In the striatum, different astrocyte populations differentially regulate goal-directed behavior. Habitual behavior can become detrimental when the reward or goal has changed. Chemogenetic activation of dorsomedial striatum astrocyte Gq signaling promotes flexible goal-directed behavior in an operant paradigm in which control mice without astrocyte activation continued to demonstrate habitual behavior (Kang et al., 2020). Astrocyte Gq activation differentially affected the activity of local indirect and direct pathway medium spiny neurons (MSNs). In this same study, mice lacking ENT1, an astrocyte adenosine transporter, did not transition from habitual to goal-directed behavior indicating a role for astrocyte adenosine in behavioral flexibility. Astrocyte regulation of extracellular glutamate levels, by the glutamate transporter EAAT2 (alias GLT-1), is also implicated in behavioral flexibility (Boender et al., 2021). EAAT2 expression rises in dorsolateral striatal astrocytes with increased habitual behavioral inflexibility in an operant paradigm. Yet, chemogenetic activation of astrocyte Gq signaling during overtraining in the operant paradigm reduces EAAT2 expression and restores behavioral flexibility. Thus, neuron-astrocyte interactions underly aspects of behavioral flexibility.

Striatal astrocytes are also implicated in attention. Chemogenetic activation of astrocyte Gi-GPCR pathways, mimicking endogenous GABA_B_ agonism, increases excitatory synapse number, MSN firing rate, and hyperactivity behavior in the open field assay. Further, attention is disrupted in both a modified open field assay and novel object recognition test (Nagai et al., 2019). These synaptic and behavioral effects were reversible and returned to control levels 48 hours post-CNO injection. Astrocyte Gi activation through hM4Di DREADD also increased astrocyte thrombospondin-1 and blockade of the thrombospondin-1 receptor rescues synaptic and behavioral deficits (Nagai et al., 2019). The authors propose a model in which MSN-released GABA induces astrocyte release of the synaptogenic cue thrombospondin-1, inducing synapse formation and increasing excitatory transmission from MSNs resulting in behavioral alterations. These findings suggest that astrocyte secreted thrombospondin-1 can affect behavior over a short timescale, highlighting the ability of astrocyte secreted factors to affect behavior, either independently of, or alongside, gliotransmission (Nagai et al., 2019). Striatal astrocytes are further implicated in repetitive self-grooming behavior. Reduction of striatal astrocyte calcium signaling through overexpression of a modified calcium plasma membrane pump (CalEx) altered local MSN activity and increased self-grooming without affecting motor ability or anxiety-like behavior (Yu et al., 2018).

Lastly, astrocytes are implicated in reward and addiction behavior. Studies have employed astrocyte Gq-GPCR activation to query the role of astrocytes in ethanol intake. Activation of PFC astrocytes increased ethanol consumption in ethanol-naïve male mice whereas reduction of astrocyte calcium via CalEx expression reduced ethanol drinking indicating a bidirectional regulation of ethanol-consumption by PFC astrocytes (Erickson et al., 2021). In the basolateral amygdala, activation of astrocytes reduced ethanol consumption in male mice (Nwachukwu et al., 2021). Likewise, in the nucleus accumbens (NAc), a key reward center, activation of astrocytes in male rats decreased ethanol-seeking behavior after abstinence (Bull et al., 2014). Further, activation of NAc astrocytes in female mice reduced reward-seeking behavior in ethanol dependent mice (Giacometti et al., 2020). Ethanol exposure alters gene expression in astrocytes in a sex-specific manner (Wilhelm et al., 2016) however, none of the above studies tested both male and female mice making direct comparisons of behavior difficult. Future studies are required to identify if astrocytes contribute to sex-specific ethanol-use and reward behavior.

NAc Gq-GPCR activation in male rats also reduced cue-induced reinstatement of cocaine seeking but did not affect initial cocaine self-administration indicating a potential role for astrocytes in relapse behavior (Scofield et al., 2015). Generally, NAc astrocyte activity correlates with reduced substance seeking and reduced relapse. Recently, Corkrum et al. (2020) demonstrated that NAc astrocytes respond to dopamine with increased calcium signaling, a response augmented by amphetamine, and absent in *Itpr2* knockout mice or in the presence of G protein signaling blockade. Chemogenetic Gq-GPCR activation of NAc astrocytes depresses local excitatory neuronal transmission, via adenosine A1 receptor signaling. Further, *Itpr2* knockout mice and mice lacking dopamine D1 receptors in astrocytes showed reduced amphetamine-induced locomotor activity (Corkrum et al., 2020) indicating a role for astrocytes in the behavioral effects of amphetamine and dopaminergic circuitry. Therefore NAc astrocytes regulate reward and addiction behavior, in part, through dopamine responsivity. Future studies may examine astrocytes as a therapeutic target for addiction treatment.

## Challenges and Future Prospects

In this review we have discussed the role of astrocytes in regulation of behavior. Acute manipulation of astrocytes with chemogenetic and optogenetic tools allow for the probing of causal relationships between astrocyte activity and animal behavior. This is in contrast to earlier gene knockout, or even inducible knockout studies, that relied on the manipulation of astrocytes over a timescale that might allow for compensatory mechanisms to affect circuitry, neuronal networks, and downstream behavior. Indeed, the ability of astrocytes to regulate synapses extends to larger circuitry and behavioral outputs. Future studies of neural circuits and behavior must therefore consider astrocyte contributions and may even incorporate astrocytes into their circuit diagrams or computational models. While not discussed here, it will also be important to consider the impact of other brain cells such as microglia (Yirmiya and Goshen, 2011; Werneburg et al., 2017), oligodendrocytes (Suminaite et al., 2019; Xin and Chan, 2020), and vascular cells (Filosa et al., 2016; Stackhouse and Mishra, 2021) on neuronal and astrocyte activity, and ultimately behavior.

Toward further understanding how astrocyte gliotransmission affects local circuitry and behavior *in vivo*, there is a growing list of neurochemical sensors that may provide insight (Looger and Griesbeck, 2012; Scofield et al., 2015; Beyene et al., 2019; Leopold et al., 2019). Many studies reviewed here focused on astrocyte interactions with local neuronal circuitry, as would be expected given that astrocytes do not project to other brain regions as neurons do. However, future studies, and the tools used for experiments, must consider the syncytium of astrocytes connected via astrocyte gap junctions (Landis and Reese, 1982) and the potential spreading of any manipulation within this network. Further, astrocyte gene expression changes across development and into adulthood (Boisvert et al., 2018) therefore it remains critical to consider developmental timing in choice of tool for behavioral studies.

### Expanding the Genetic Toolbox for Probing Astrocyte Heterogeneity and Function

The currently used Aldh1l1 (Tsai et al., 2012), GFAP (Lee et al., 2008), and GfaABC1D (Shigetomi et al., 2013; Figure 2) promoters efficiently target astrocytes but, in some cases, may target non-astrocyte cells such as the tanycytes lining the ventricle (Chen et al., 2016) or subsets of neurons (Cahoy et al., 2008; Farhy-Tselnicker et al., 2017). Numerous recent papers highlight the transcriptional diversity of astrocytes (Cahoy et al., 2008; Zhang et al., 2014; Srinivasan et al., 2016; Chai et al., 2017; Boisvert et al., 2018; Clarke et al., 2018; Batiuk et al., 2020; Bayraktar et al., 2020; Farhy-Tselnicker et al., 2020; Guttenplan et al., 2020; Pan et al., 2020). These databases of astrocyte gene expression may identify new astrocyte specific genetic markers for astrocyte targeting. Further, they may allow for the functional interrogation of astrocyte subtypes, either through genetic markers specific to astrocyte subtypes or through intersectional genetics using existing astrocyte Cre-dependent tools paired with new subtype-specific Flp-dependent tools (Figure 2B; Dymecki et al., 2010; Fenno et al., 2014). Already there is evidence for a small population of genetically distinct astrocytes serving an important behavioral role. The OTR-expressing subtype of central amygdala astrocytes makes up around 19% of all astrocytes in that brain region yet mediates the anxiolytic effects of oxytocin (Wahis et al., 2021a). Future experiments should address the behavioral role of the remaining central amygdala astrocytes as well as exploring astrocyte subtype influences on behavior in other brain regions. If a local astrocyte subtype mediates one behavioral function, might a nearby subtype mediate an opposing behavioral function? Following identification of functional astrocyte subtypes, the next step will be to probe their interactions during naturalistic behaviors. Such experiments will be technically challenging and will likely require two photon imaging with multicolor calcium indicators paired with advanced ethologically relevant behavior tracking (Mathis et al., 2018; Wiltschko et al., 2020). As noradrenaline exerts behavioral state-specific effects on large populations of astrocytes (Paukert et al., 2014), an additional challenge will be to understand how more global astrocyte activity resulting from neuromodulation interacts with local circuit- or subtype-specific astrocyte activity (Bazargani and Attwell, 2016).

### Increasing Understanding of Astrocyte Endogenous Signaling Mechanisms

Many of the tools discussed here target astrocyte GPCRs ultimately leading to increased calcium signaling (Yu et al., 2020). Key questions toward resolving astrocyte contributions to behavioral regulation include, (1) what additional intracellular signaling pathways are involved, (2) how is astrocyte gene expression affected, and (3) how does spatially localized astrocyte calcium activity influence behavior? Signaling pathways following GPCR activation in astrocytes are not fully resolved and should be the focus of future investigations. Understanding these molecular mechanisms will also inform tool design for physiologically relevant methods to manipulate astrocytes and probe involvement in behavioral regulation. Future studies should also address how GPCR signaling in astrocytes may alter gene expression. RNA sequencing following GPCR activation can reveal changes in gene expression that may underlie astrocyte regulation of synapses, circuits, and behavior. For example, how Gi-GPCR activation of striatal astrocytes mechanistically increases hyperactivity and attention was revealed through RNA sequencing of astrocytes following acute Gi-GPCR activation compared to control animals, revealing higher expression of the synaptogenic cue thrombospondin-1 (Nagai et al., 2019). As astrocyte processes display spontaneous and spatially localized calcium transients that can occur independently of calcium release from the endoplasmic reticulum (Shigetomi et al., 2010; Di Castro et al., 2011; Khakh and McCarthy, 2015; Bazargani and Attwell, 2016; Rungta et al., 2016; Agarwal et al., 2017), future studies should probe the involvement of spatially localized astrocyte calcium activity in behavior. For example, SpiCee is a recently developed genetically encoded calcium chelator designed to manipulate intracellular calcium levels within different subcellular regions (Ros et al., 2020) which may prove useful for such studies. Research on astrocyte intracellular signaling pathways, gene expression, and calcium localization will continue to inform on how astrocytes shape behavior.

### Sex Differences in Astrocytes and Behavior

Neuroscience research in mice has regularly focused on male subjects with one analysis finding only 20% of studies used both male and female mice while around 25% did not state the sex of subjects (Beery and Zucker, 2011; Prendergast et al., 2014). Cortical astrocytes show significant sex differences in gene expression during development and astrocytes express estrogen receptors (Wright et al., 2010; Rurak et al., 2020). As noted above, ethanol consumption has sex-specific effects on gene expression in astrocytes (Wilhelm et al., 2016) indicating the potential for environmental factors to effect astrocyte function in sex-specific ways. Recently, astrocyte-derived thrombospondin-1 was reported to induce cortical synaptogenesis in a sex-specific manner (Mazur et al., 2021). Taken together, sex differences in astrocyte regulation of behavior are likely but remain unexplored. Future studies should employ both male and female mice to further elucidate sex differences in astrocytes. As astrocytes also contribute to pathological behavior in disordered states (Dallerac and Rouach, 2016; Santello et al., 2019), the use of male and female mice in astrocyte research will have important implications for human health (Beery and Zucker, 2011).

## Conclusion

**FIGURE 3:**
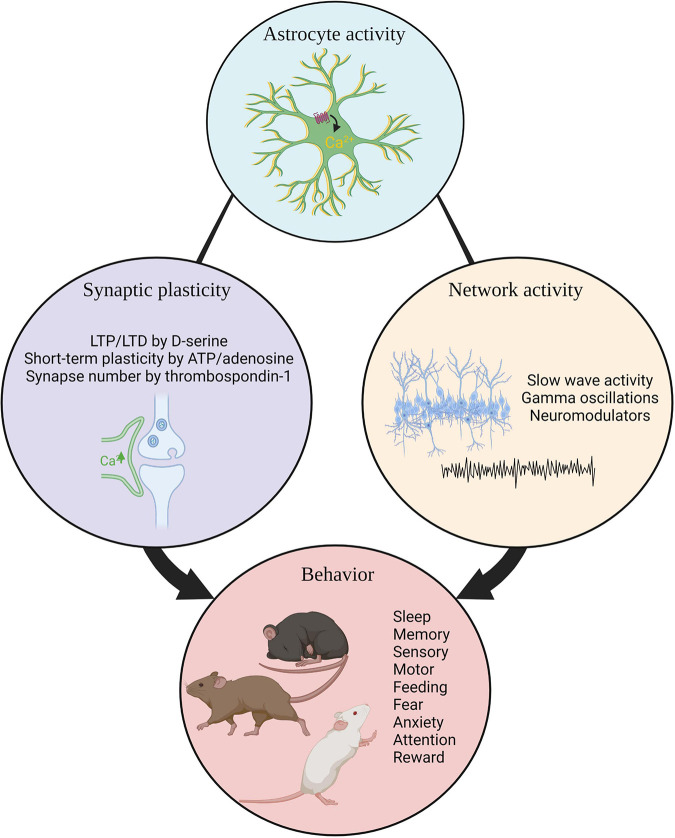
Summary. Astrocyte activity influences synaptic plasticity, neuronal network activity, and organismal behavior. LTP, long term potentiation.; LTD, long term potentiation.

Research reviewed here demonstrates the importance of astrocytes in the regulation of homeostatic functions and diverse behavioral processes. Astrocytes can influence local neuronal circuitry through release of secreted factors or gliotransmitters leading to changes in synaptic structure and altered neuronal excitability. Through these capacities, astrocytes are able to shape behavior via changes in neuronal activity (Figure 3). In turn, neuronal activity can influence astrocyte activity thus these interactions are bidirectional. The majority of studies reviewed here parsed astrocyte behavioral function in circuits known to modulate a specific behavioral function. Acute manipulation of hypothalamic astrocytes influences feeding (Yang et al., 2015; Chen et al., 2016), hippocampal astrocytes impact memory (Adamsky et al., 2018; Mederos et al., 2019, 2021; Kol et al., 2020), and amygdala astrocytes regulate fear (Martin-Fernandez et al., 2017). These functions are already known to be subserved by those brain regions and now astrocyte contributions are beginning to be appreciated. Future studies may reveal surprising roles for astrocytes in behavioral regulation, independent of known neuronal circuit functions. As understanding of astrocyte heterogeneity increases, future experiments will address whether astrocytes perform subtype-specific functions underlying neuronal circuit activity and organismal behavior.

## Author Contributions

Both authors conceptualized and wrote this review together. Both authors contributed to the article and approved the submitted version.

## Conflict of Interest

The authors declare that the research was conducted in the absence of any commercial or financial relationships that could be construed as a potential conflict of interest.

## Publisher’s Note

All claims expressed in this article are solely those of the authors and do not necessarily represent those of their affiliated organizations, or those of the publisher, the editors and the reviewers. Any product that may be evaluated in this article, or claim that may be made by its manufacturer, is not guaranteed or endorsed by the publisher.
